# Extension of Operating Range in Hybrid Cascaded H-Bridge Inverters with Capacitor Voltage Balancing Capability

**DOI:** 10.3390/s24030991

**Published:** 2024-02-03

**Authors:** Abhilash Tirupathi, Jonathan Pribadi, Min-Seok Kim, Dong-Choon Lee

**Affiliations:** Department of Electrical Engineering, Yeungnam University, Gyeongsan 38541, Gyeongbuk, Republic of Korea; tabhilash@yu.ac.kr (A.T.); jonathanpribadi@ynu.ac.kr (J.P.); lupinsuk@ynu.ac.kr (M.-S.K.)

**Keywords:** capacitor voltage balancing, hybrid cascaded H-bridge (HCHB) inverters, reduced device count, multilevel converters, operating range extension

## Abstract

In this article, a generalized control scheme is proposed to extend the operating range of three-phase hybrid cascaded H-bridge (HCHB) inverters into various voltage levels without necessitating alterations to the core structure or the integration of additional H-bridge submodules. This study addresses a critical challenge related to capacitor voltage drift at various modulation indices and power factors, which is a serious impediment to various applications. To overcome this challenge, a novel balancing control scheme has been developed based on the injection of two independent offset voltages to simultaneously control the DC-link and flying capacitors. A distinctive aspect of the proposed technique involves adjusting the common reference voltage to attain the nearest level in the same cluster, thereby mitigating the insufficiency of redundant switching states. The effectiveness of the proposed technique to regulate the capacitor voltages at various operating conditions has been verified through simulation and experimental results.

## 1. Introduction

Over the past few decades, extensive studies have been widely carried out to meet the increased market demand for high-power conversion systems, such as industrial motor drives, high-voltage direct-current (HVDC) transmission, grid-tied energy storage, uninterruptible power supplies (UPS), and renewable energy systems [1,2,3,4,5,6,7,8]. Multilevel converters have emerged as the preferred choice in systems spanning medium to high voltage ratings due to the inherent advantages, such as lower voltage stress at switch devices, reduced harmonic distortion, higher power quality, improved scalability, fault tolerability, and diminished electro-magnetic interference (EMI) compared to two-level counterparts [2,9]. Nevertheless, the number of components in the classical multilevel topologies, such as diode-clamped inverters, flying capacitor inverters, cascaded H-bridge inverters, T-type inverters, generalized P2 inverters, and multilevel active-clamped inverters, increases in proportion to the number of levels [4,10,11,12,13,14,15,16,17,18,19,20,21,22,23].

In order to reduce the number of semiconductor switches, several topologies have been proposed with multiple isolated DC voltage sources, which provide a modular and scalable approach to designing power converters for various voltage levels [24,25,26]. However, these isolated DC voltage sources are provided through the integration of additional transformers in conjunction with front-end rectifiers. These additional components are often deemed less favorable in light of the heightened complexity, augmented cost, and substantial challenges in terms of control and reliability, thereby contributing to the limited acceptance of these configurations. Consequently, the quest for converters which are capable of mitigating these drawbacks while maintaining a low device count has led to the development of numerous recent topologies.

One of the most established and extensively researched topologies is the five-level hybrid active neutral-point-clamped (5L-ANPC) inverter, which is an extension of the three-level (3L-ANPC) inverter with an additional flying capacitor (FC) unit at each phase [27,28,29]. This topology provides sufficient redundant switching states, which ease the capacitor voltage control and improve the output voltage quality compared to the three-level predecessors. However, the necessity for additional FC units for voltage level extension results in an increase in converter volume.

Another variant of the ANPC family is the four-level hybrid-clamped (4L-HC) inverter. This topology stems from the aforementioned 5L-ANPC inverter with an additional middle DC-link capacitor. This structure provides a number of benefits such as uniform voltage rating at each switch and uniform voltages at the split DC-link and flying capacitors. In addition, the five-level and six-level variants of this topology, namely hybrid flying capacitor (HFC) inverters, have also been developed with resembling structures [30,31,32,33,34]. However, despite the improvement of voltage quality, all of these inverters still exhibit the aforementioned drawback, i.e., they require additional components upon voltage level extension.

Other hybrid topologies such as nested multilevel converters and stacked multi-cell converters have become alternatives to the ANPC family. These topologies have proven to enhance voltage quality by incorporating one or two additional voltage levels in each phase. This is accomplished by introducing commutation cells between the primary inverter structure and the DC-bus capacitors. However, these voltage level extensions come at the cost of either a significantly increased device count or more complicated capacitor balancing control [35,36,37,38,39,40,41,42,43].

The integration of switched-capacitor (SC) circuits into multilevel inverters has also gained prominence in the last decade. These circuits employ capacitors, power switches, and/or diodes to convert a fixed DC-link voltage into multilevel voltage, enabling inductorless and transformerless operation with voltage-boosting capabilities and inherent capacitor self-voltage balancing. Although SC integration in DC-DC converters has been extensively studied and commercialized, the application to multilevel AC-voltage generation is still a growing area of research [44,45,46,47,48]. There are considerations related to component count, complexity, and the large value of current stress imposed by the input DC source which limit the expected power range of these converters.

Lately, there has been a surge of hybrid topologies stemming from diverse multilevel structures and cascaded H-bridge (CHB) submodules, where the primary goal is to generate seven or more levels at each phase leg [3,49,50]. These inverters possess the versatility to scale up to higher voltage levels by modifying the core multilevel structures or by incorporating additional CHB submodules. For instance, a nine-level inverter can be obtained from the combination of a 3L-ANPC inverter with two H-bridge submodules and the combination of a 5L-ANPC with one H-bridge submodule. However, it is important to note that these voltage-level extensions come with an increase in switches and capacitor count, and thus lead to a substantial uptick in the overall volume.

In order to maintain structure simplicity, a more streamlined approach has been taken by combining a three-level cascaded inverter and a CHB submodule at each phase leg [51,52,53]. Through the selective design of DC-bus and flying capacitor voltages, this hybrid cascaded H-bridge (HCHB) structure eliminates the necessity of modifying the core structure or adding more H-bridge submodules for synthesizing a higher voltage level. However, this approach minimizes the device count at the cost of a constrained operating range, where capacitor voltage drifts are expected to take place at various modulation indices and power factors. This issue has hampered the competitiveness of these topologies and become an impediment to widespread adoption across various applications.

In this article, a novel control scheme is presented to overcome operational constraints in various HCHB inverters. The key components which affect capacitor voltage drifts have been analyzed to construct a generalized operating scheme for inverters with various numbers of levels. Analogous to conventional approaches, a proportional offset is injected to the reference voltages to counter the voltage deviation at DC-link capacitors. In order to maintain voltage balance at each flying capacitor and compensate for the lack of redundant switching states, the proposed technique forces the common reference voltage to attain the nearest level within the same cluster which possesses the opposite charging characteristic. With this method, each HCHB inverter can be operated across the complete spectrum of modulation indices and power factors. The effectiveness of the proposed technique is verified through various simulation results and validated in experiments with a downscaled prototype.

## 2. Overview of HCHB Inverters

In this section, the topological properties and basic operating scheme of HCHB inverters with various number of levels are discussed.

### 2.1. Circuit Configuration

**Figure 1:**
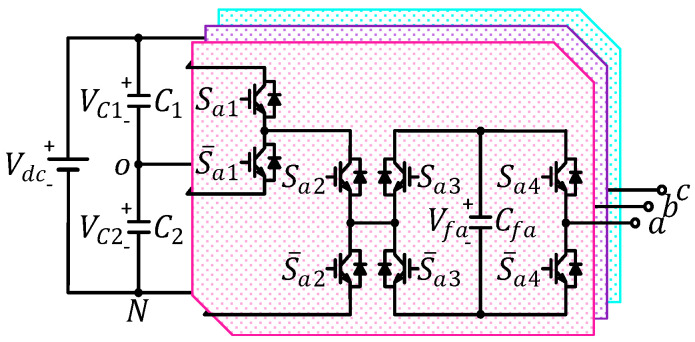
Generalized structure of three−phase HCHB inverters.

The generalized structure of three-phase hybrid cascaded H-bridge (HCHB) inverters is illustrated in Figure 1. This topology consists of two split DC-link capacitors ($C_{1}$ and $C_{2}$), one flying capacitor ($C_{fx}$), and four pairs of complementary switches at each phase leg (x: a, b, or c).

**Figure 2:**
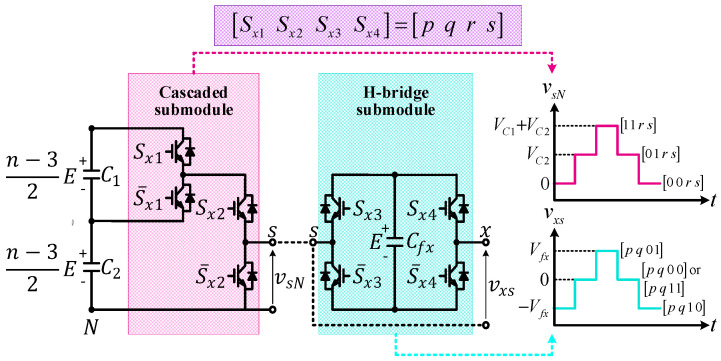
Voltage synthesis for *n*−level HCHB inverters according to switching patterns at cascaded and H-bridge submodules.

When each phase leg is seen as the combination of one cascaded submodule and one H-bridge submodule, as depicted in Figure 2, the synthesis of output voltage at every phase is defined as follows:

(1) $$v_{xN}=\left(S_{x1}\right)\left(V_{C1}\right)+\left(S_{x2}\right)\left(V_{C2}\right)+\left(S_{x4}-S_{x3}\right)\left(V_{fx}\right),$$

where $v_{xN}$, $S_{x1}$, $S_{x2}$, $S_{x3}$, and $S_{x4}$ denote the output voltage at each phase leg and the switching status of every semiconductor at phase leg.

### 2.2. Generalized Switching Pattern

The combination of both cascaded inverter and H-bridge submodules generates up to 12 switching states, as listed in Table 1, where the direction of current flowing through each of the split DC-link and flying capacitors ($i_{C1x}$, $i_{C2x}$, and $i_{fx}$) varies according the polarity of the output current ($i_{x}$). Since each of the submodule output voltages, namely $v_{sN}$ and $v_{xs}$, generates three levels of voltage, the maximum and minimum numbers of levels generated at each phase leg are nine and five, respectively.

#### 2.2.1. Voltage Synthesis for Five-Level Inverter

In order to generate five-level output voltage at every phase, the capacitor voltages should be configured in such a way that switching events at H-bridge submodules only contribute two additional switching states to the available three switching states generated by cascaded submodules. This is achieved by constructing the capacitor voltages as follows:

(2) $$V_{C1}=V_{C2}=V_{fx}=E=V_{dc}/2,$$

where $V_{C1}$, $V_{C2}$, $V_{fx}$, $V_{dc}$, and E are the voltages of split DC-link capacitors and flying capacitor, the DC-bus voltage, and base voltage of the inverter. Therefore, the maximum amplitude of pole voltage ($v_{xo}$) is 2E or $V_{dc}$, which is two times as high as that of the conventional buck inverters ($V_{dc}/2$). Meanwhile, every switch sustains the same voltage stress at $V_{dc}/2$.

#### 2.2.2. Voltage Synthesis for Seven-Level Inverter

Synthesis of a higher number of levels requires an adjustment of the split DC-link capacitor voltages, whereas those of the flying capacitors are maintained at the base voltage to keep the total capacitor voltages low. In order to achieve seven-level output voltage at every phase, the capacitor voltages should be controlled as follows:

(3) $$\left\{\begin{array}{c} V_{C1}=V_{C2}=2E=V_{dc}/2\\ V_{fx}=E=V_{dc}/4 \end{array}\right.,$$

The maximum amplitude of pole voltage is 3E or $\left(3/4\right)V_{dc}$, which is 1.5 times as high as that of the conventional buck inverters. Meanwhile, the switches at the cascaded and H-bridge submodules should sustain the voltage stress at $V_{dc}/2$ and $V_{dc}/4$, respectively.

#### 2.2.3. Voltage Synthesis for Nine-Level Inverter

In order to achieve nine-level output voltage, which is the maximum level available for this inverter structure, the capacitor voltages should be regulated in such a way that all switching states should be designed to generate unique $v_{xN}$ without any overlapping voltages. Therefore, the capacitor voltages are arranged as follows:

(4) $$\left\{\begin{array}{c} V_{C1}=V_{C2}=3E=V_{dc}/2\\ V_{fx}=E=V_{dc}/6 \end{array}\right..$$

With this configuration, the maximum amplitude of pole voltage is 4E or $\left(2/3\right)V_{dc}$, which is 1.33 times as high as that of the conventional buck inverters. The voltage stresses of switches at the cascaded and H-bridge submodules are $V_{dc}/2$ and $V_{dc}/6$, respectively.

#### 2.2.4. Generalized Voltage Synthesis for HCHB Inverters

Each of the HCHB configurations discussed above generates odd-level output voltage. As a general pattern, the capacitor voltages are formulated as follows:

(5) $$\left\{\begin{array}{c} V_{C1}=V_{C2}=\left(0.5\right)\left(n-3\right)E\\ V_{fx}=E=V_{dc}/\left(n-3\right) \end{array}\right.,$$

where n denotes the number of levels. For odd-level inverters, the value of each split DC-link capacitor is always an integer which is multiplied with the base voltage. Meanwhile, the multiplier value for even-level inverters is always a non-integer, which results in non-uniform voltage steps at the staircase waveforms. To address this issue, the values of both $V_{C1}$ and $V_{C2}$ should be rounded up and down to the nearest integers in such a way that the sum is still equal to $\left(n-3\right)E$. Therefore, each of these even-level inverters can be constructed with two configuration options, as given in Table 2. Note that the pole voltages of even-level inverters exhibit asymmetric waveforms due to the uneven voltage distribution at the split DC-link capacitors.

Since the voltages of split DC-link capacitors of most HCHB inverters are higher than those of the flying capacitors, the voltage stress of each switch in the cascaded submodule is also higher than those of the H-bridge submodule. In addition to producing lower voltage steps (dv/dt) at each phase leg, the inverters with a higher number of levels also exhibit lower total standing voltages (TSV) due to the lower voltage stress at H-bridge submodules, as listed in Table 2.

### 2.3. Operating Range Limits

**Figure 3:**
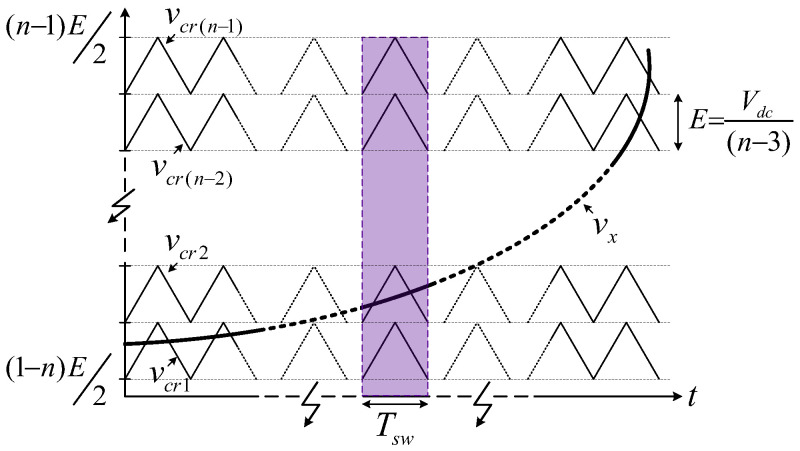
In-phase disposition level-shifted multicarrier PWM (IPD-LSPWM) for *n*-level HCHB inverters.

In the previous study [52], a three-phase 7L-HCHB inverter was developed and operated with an in-phase disposition level-shifted multicarrier PWM (IPD-LSPWM), as illustrated in Figure 3. In this technique, the voltage reference at each phase leg is compared with the carrier waveforms ($v_{cr1}–v_{cr(n-2)}$) to determine the switching of each device [54].

Suppose that the voltage and current at each phase leg for various n-level inverters, which are denoted by $v_{x}$ and $i_{x}$, are defined as follows:

(6) $$v_{x}=U_{x}\sin\left(\omega t+\delta_{x}\right)=\frac{m_{a}\left(n-1\right)V_{dc}}{\left(2n-6\right)}\sin\left(m_{f}\omega_{0}t+\delta_{x}\right),$$

(7) $$i_{x}=I_{x}\sin\left(m_{f}\omega_{0}t+\delta_{x}+\varphi \right),$$

where $U_{x}$, $I_{x}$, ω, $m_{a}$, $m_{f}$, $\omega_{0}$, $\delta_{x}$, and φ denote the amplitudes of pole voltage and output current, operating frequency, amplitude and frequency modulation indices, fundamental angular frequency, initial phase angle, and the phase angle between $v_{x}$ and $i_{x}$, respectively.

Since the staircase voltages are formed by the DC-link and flying capacitor voltages, the voltage balance of each capacitor needs to be maintained. The operating range limitation in seven-level inverters has been analyzed by deducing the accumulated charge variation in each flying capacitor during half of the fundamental cycle, where it has been concluded that these topologies can be operated only at $m_{a}\leq 0.82$. Meanwhile, the split DC-link capacitor voltages are theoretically capable of self-balancing due to the symmetry in the polarity of phase current over one fundamental cycle. Nevertheless, the implementation of closed-loop control for voltage balancing is necessary to mitigate voltage drifts in the real environment with dynamic conditions [50,51,52].

**Figure 4:**
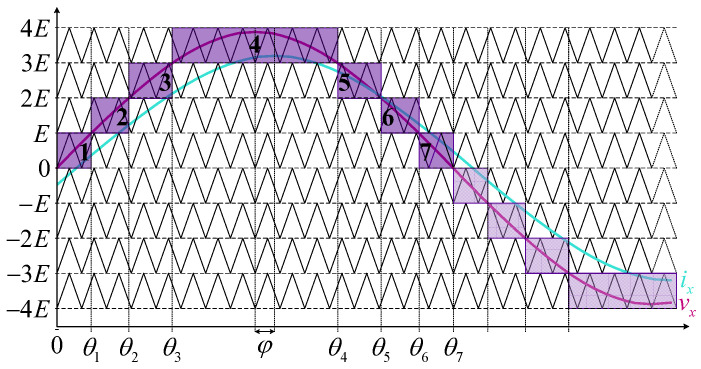
Output voltage and current at each phase leg of 9L-HCHB inverter during a half of the fundamental cycle.

A similar approach can be applied to analyze the balancing capability of any n-level HCHB inverter. For instance, the output voltage and current of a 9L-HCHB inverter are illustrated in Figure 4, where the staircase waveform during half of the fundamental cycle can be divided into seven areas. The demarcation angle for each area can be calculated based on the geometrical correlation as follows:

(8) $$\theta_{i}=\left\{\begin{array}{cc} \frac{T_{0}}{2\pi }\sin^{-1}\left(\frac{iE}{U_{x}}\right), & 1\leq i\leq \frac{n-3}{2}\\ \frac{T_{0}}{2\pi }\left(\pi -\sin^{-1}\left(\frac{\left(n-2-i\right)E}{U_{x}}\right)\right), & \frac{n-1}{2}\leq i\leq n-2 \end{array}\right.,$$

where $\theta_{i}$ and $T_{0}$ denote the final demarcation angle of area i and the period of fundamental wave, respectively.

The accumulated charge variation in each area can be calculated as follows:

(9) $$Q_{tot,x}=\sum_{i=1}^{n-2}Q_{FCi,x}=\sum_{i=1}^{n-2}\left(\int_{\theta_{i-1}}^{\theta_{i}}{\bar{i}}_{FCi,x}\right),$$

where $Q_{FCi,x}$, $Q_{tot,x}$, and ${\bar{i}}_{FCi,x}$ denote the charge variation at area i, total charge variation during half of the fundamental cycle, and the average current that flows into the flying capacitor at area i. The value of ${\bar{i}}_{FCi,x}$ is calculated according to the conduction path in area i in correlation with the output current. For instance, charge variations in 9L-HCHB inverter are obtained as follows:

(10) $$Q_{FC,1x}=\int_{0}^{\theta_{1}}{\bar{i}}_{FC1,x}dt=\int_{0}^{\theta_{1}}-\frac{U_{x}\sin\left(\omega t\right)}{E}I_{x}\sin\left(\omega t+\varphi \right)dt,$$

(11) $$Q_{FC,2x}=\int_{\theta_{1}}^{\theta_{2}}{\bar{i}}_{FC2,x}dt=\int_{\theta_{1}}^{\theta_{2}}\left(\frac{2U_{x}\sin\left(\omega t\right)}{E}-3\right)I_{x}\sin\left(\omega t+\varphi \right)dt,$$

(12) $$Q_{FC,3x}=\int_{\theta_{2}}^{\theta_{3}}{\bar{i}}_{FC3,x}dt=\int_{\theta_{2}}^{\theta_{3}}\left(3-\frac{U_{x}\sin\left(\omega t\right)}{E}\right)I_{x}\sin\left(\omega t+\varphi \right)dt,$$

(13) $$Q_{FC,4x}=\int_{\theta_{3}}^{\theta_{4}}{\bar{i}}_{FC4,x}dt=\int_{\theta_{3}}^{\theta_{4}}\left(3-\frac{U_{x}\sin\left(\omega t\right)}{E}\right)I_{x}\sin\left(\omega t+\varphi \right)dt,$$

(14) $$Q_{FC,5x}=\int_{\theta_{4}}^{\theta_{5}}{\bar{i}}_{FC5,x}dt=\int_{\theta_{4}}^{\theta_{5}}\left(3-\frac{U_{x}\sin\left(\omega t\right)}{E}\right)I_{x}\sin\left(\omega t+\varphi \right)dt,$$

(15) $$Q_{FC,6x}=\int_{\theta_{5}}^{\theta_{6}}{\bar{i}}_{FC6,x}dt=\int_{\theta_{5}}^{\theta_{6}}\left(\frac{2U_{x}\sin\left(\omega t\right)}{E}-3\right)I_{x}\sin\left(\omega t+\varphi \right)dt,$$

(16) $$Q_{FC,7x}=\int_{\theta_{6}}^{\theta_{7}}{\bar{i}}_{FC7,x}dt=\int_{\theta_{6}}^{\theta_{7}}-\frac{U_{x}\sin\left(\omega t\right)}{E}I_{x}\sin\left(\omega t+\varphi \right)dt.$$

A conclusion regarding the capacitor balancing capability can be obtained by summing up the entire charge variations, where $Q_{tot,x}$ must be positive to maintain the capacitor charge balance under IPD-LSPWM.

**Figure 5:**
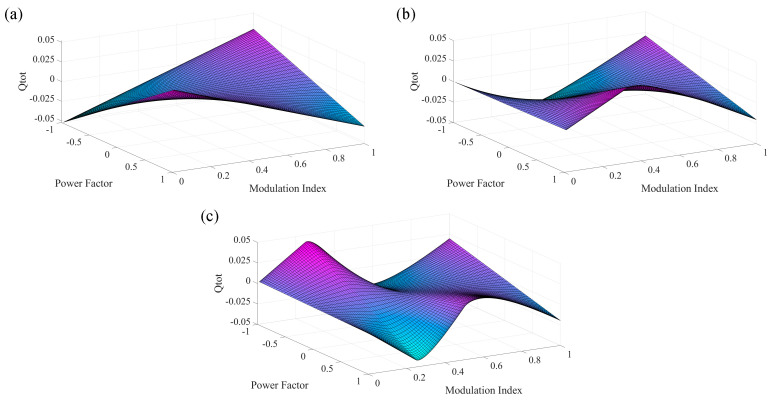
Accumulated charge variation in each flying capacitor during half of the fundamental cycle at various modulation indices and power factors. (**a**) 5L−HCHB. (**b**) 7L−HCHB. (**c**) 9L−HCHB.

In order to observe the operating range limit of various HCHB inverters, the values of $Q_{tot,x}$ have been calculated and plotted in Figure 5 by assuming that the fundamental operating frequency and output current amplitude are set at $f_{0}$ = 60 Hz and $I_{x}$ = 10 A.

**Figure 6:**
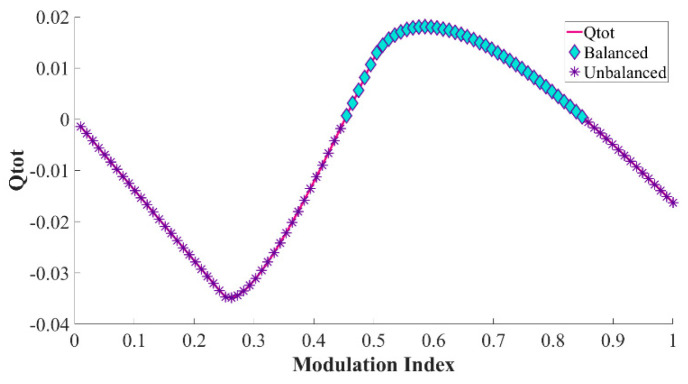
Accumulated charge variation in each flying capacitor of 9L−HCHB inverter at PF = 0.83.

Note that $I_{x}$ only determines the amplitude of $Q_{tot,x}$, whereas the polarity remains identical. Since the value of $Q_{tot,x}$ in each graph is not always positive, voltage drifts are expected to take place in any n-level HCHB inverter when it is operated at various modulation indices and power factors. For instance, consider the charge variation in the flying capacitors of 9L-HCHB inverter for cos (φ) = 0.83, as illustrated in Figure 6. In this case, the value of $Q_{tot,x}$ is negative at $m_{a}<$ 0.45 and $m_{a}>$ 0.84, where voltage imbalance is expected to occur. Such operating range limits adversely negate the benefit this structure offers, i.e., flexibility to increase the number of levels, and thus diminish the competitiveness of this topology.

A number of control balancing techniques that are commonly used in various multilevel inverters have been listed in Table 3. Despite the effectiveness, none of these techniques satisfy the requirement of capacitor voltage balancing in the HCHB inverters without adversely affecting the output voltage quality, increasing the computational burden, or increasing the device count of the inverter.

## 3. Proposed Balancing Control Scheme

In the previous section, the problem with operating range limit has been discussed. In order to solve this issue, a novel control is proposed by injecting two separate offsets into the reference voltage at each phase ($v_{x,ref}=v_{x}/E$).

### 3.1. Control of DC-Link Capacitor Voltages

The structure of HCHB inverters position the DC-link capacitor voltages as significant contributors to the formation of pole voltage at each phase leg. Since the values of $V_{C1}$ and $V_{C2}$ should be regulated at $\left(0.5n-1.5\right)E$ for odd-level inverters and the nearest integers for even-level inverters, a simple control scheme has been modified to maintain the error of either $V_{C1}$ or $V_{C2}$ within the allowable range. Table 1 shows how the switching states in any n-level HCHB inverter provide similar conduction paths to discharge the voltages of $C_{1}$ and $C_{2}$ as the reference voltage is controlled at certain levels. In order to control these capacitor voltages, a proportional offset voltage is injected to $v_{x,ref}$ as follows:

(17) $${\left[\begin{array}{cc} \Delta V_{C12} & \Delta V_{C12}^{*} \end{array}\right]}^{T}=\left[\begin{array}{c} V_{C1}-V_{C2}\\ V_{C1}^{*}-V_{C2}^{*} \end{array}\right],$$

(18) $$\Delta v_{offs,DC}=\left\{\begin{array}{cc} k_{dc}\left(V_{C1}-V_{C2}\right), & \left|\Delta V_{C12}-\Delta V_{C12}^{*}\right|>\varepsilon_{DC}\left(V_{dc}/2\right)\\ 0, & \left|\Delta V_{C12}-\Delta V_{C12}^{*}\right|\leq \varepsilon_{DC}\left(V_{dc}/2\right) \end{array}\right.,$$

where ${∆V}_{C12}$ and ${∆V}_{C12}^{*}$ denote the actual and reference voltage error between $V_{C1}$ and $V_{C2}$, whereas ${∆v}_{offs,DC}$, $k_{dc}$, and $\varepsilon_{DC}$ denote the offset voltage, proportional control gain, and the maximum allowable error percentage. Typically, adhering to a general guideline, the peak-to-peak capacitor voltage ripple should be kept within ±10% of the corresponding reference [61,62].

### 3.2. Control of Flying Capacitor Voltages

As mentioned above, each flying capacitor voltage should be regulated at the base voltage ($V_{fx}^{*}=E$). Since voltage drift is expected to occur at certain modulation indices and power factors, a controller is needed to counter the deviation. Suppose that the voltage error is defined as follows:

(19) $$\Delta V_{fx}=V_{fx}-V_{fx}^{*},$$

(20) $$\left|\Delta V_{fx}\right|=\left\{\begin{array}{cc} \Delta V_{fx}, & \Delta V_{fx}\geq 0\\ -\Delta V_{fx}, & \Delta V_{fx}<0 \end{array}\right.,$$

where ${∆V}_{fx}$ denotes the calculated error between actual and reference flying capacitor voltages.

Table 1 shows the topological characteristic of HCHB inverters, where the polarity of flying capacitor current varies within half of the fundamental cycle. Since the switching state redundancy is not sufficient to correct the voltage drifts at certain modulation indices and power factors, a common offset voltage, namely ${∆v}_{offs,FC}$, should be injected to force $v_{x,ref}$ to the nearest switching states with the opposite current polarity. Since ${∆v}_{offs,FC}$ is commonly shared among the reference voltages at all phase legs, a number of common parameters should be defined as follows:

(21) $$\left|\Delta V_{f,\max}\right|=\max\left(\left|\Delta V_{fa}\right|,\;\;\left|\Delta V_{fb}\right|,\;\left|\Delta V_{fc}\right|\right),$$

(22) $${\left[\begin{array}{c} \Delta V_{f,\max}\\ i_{cm}\\ v_{cm} \end{array}\right]}^{T}=\left\{\begin{array}{cc} \left[\begin{array}{ccc} \Delta V_{fa} & i_{a} & v_{a,ref} \end{array}\right], & \left|\Delta V_{f,\max}\right|=\left|\Delta V_{fa}\right|\\ \left[\begin{array}{ccc} \Delta V_{fb} & i_{b} & v_{b,ref} \end{array}\right], & \left|\Delta V_{f,\max}\right|=\left|\Delta V_{fb}\right|\\ \left[\begin{array}{ccc} \Delta V_{fc} & i_{c} & v_{c,ref} \end{array}\right], & \left|\Delta V_{f,\max}\right|=\left|\Delta V_{fc}\right| \end{array}\right.,$$

where the value of $\left|{∆V}_{fmax}\right|$, which is obtained by sorting the absolute values of voltage errors across all phase legs, is also used to determine the common output current ($i_{cm}$) and the common voltage reference ($v_{cm}$). The value of $v_{cm}$ is classified into several clusters, each of which consists of at least two points which correspond to $v_{xN}$ with opposite charging or discharging characteristics, namely $v_{cm,C}$ and $v_{cm,D}$, respectively. If the maximum allowable error percentage for every flying capacitor voltage is denoted by $\varepsilon_{FC}$, the offset voltage should be generated whenever $\left|{∆V}_{fmax}\right|$ exceeds $\varepsilon_{FC}E$, as follows:

(23) $$\Delta v_{offs,FC}=\left\{\begin{array}{cc} 0, & \left|\Delta V_{f,\max}\right|\leq \varepsilon_{FC}E\\ v_{cm,D}-v_{cm}, & \left(\Delta V_{f,\max}-\varepsilon_{FC}E\right)i_{cm}>0\\ v_{cm,C}-v_{cm}, & \left(\Delta V_{f,\max}-\varepsilon_{FC}E\right)i_{cm}<0 \end{array}\right..$$

An example of how ${∆v}_{offs,FC}$ is obtained for a 9L-HCHB inverter is given in Table 4, where $v_{cm}$ is classified into four clusters. The value of $v_{cm}$ in cluster I corresponds to three voltage levels at $v_{xN}$, i.e., 2E, 3E, and 4E, where $v_{cm,C}=2$ and $v_{cm,D}=4$. If the capacitor voltage is overcharged during positive $i_{cm}$, the reference voltage of the phase with the highest error is forced to reach $v_{x,ref}=4$ to discharge the capacitor. Contrarily, the reference should be forced to reach $v_{x,ref}=2$ to restore the voltage of an undercharged capacitor. Note that the actions should be reversed during negative $i_{cm}$. The same principle is applied to the other clusters and other n-level CHCB inverters.

### 3.3. Summary of Proposed Balancing Scheme

**Figure 7:**
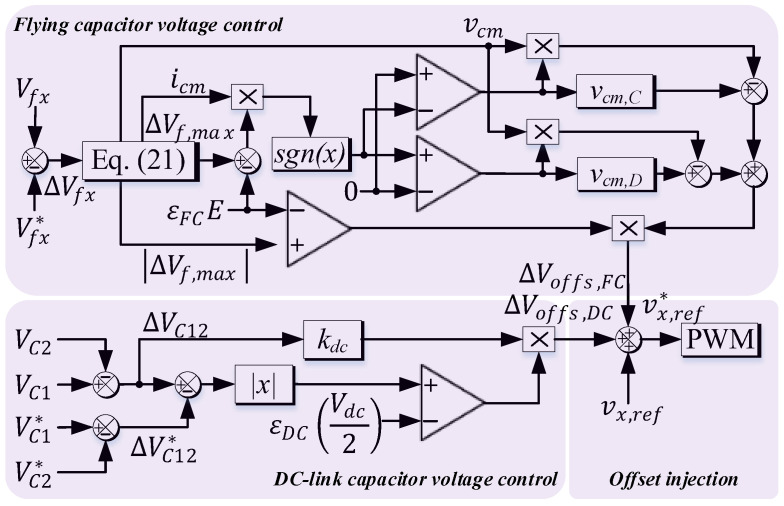
Control block diagram of HCHB inverters.

From the previous section, it can be concluded that two independent offset voltages should be generated to regulate the split DC-link and flying capacitor voltages as follows:

(24) $$\left\{\begin{array}{c} v_{x,ref}^{*}=v_{x,ref}+\Delta v_{offs}\\ \Delta v_{offs}=\Delta v_{offs,DC}+\Delta v_{offs,FC} \end{array}\right..$$

where the new voltage reference at each phase leg is denoted by $v_{x,ref}^{*}$. This new reference is compared with the normalized carrier waveforms ($v_{cr1}/E–v_{cr(n-2)}/E$) under the IPD-LSPWM technique to generate the switching signals of all devices according to the corresponding switching states in Table 1. Figure 7 shows the control block diagram of split DC-link and flying capacitor voltages. With this generalized control scheme, all capacitor voltages are regulated at the corresponding references, and thus keep the voltage THD low, particularly at HCHB inverters with a higher number of levels [63,64,65,66].

## 4. Simulation Results

In order to verify the effectiveness of this balancing control technique for various HCHB inverters, a number of simulations have been carried out for five-level, seven-level, and nine-level HCHB inverters. The parameters used for this simulation are listed in Table 5. The peak voltage ripple of each capacitor is controlled within 10% of the reference.

**Figure 8:**
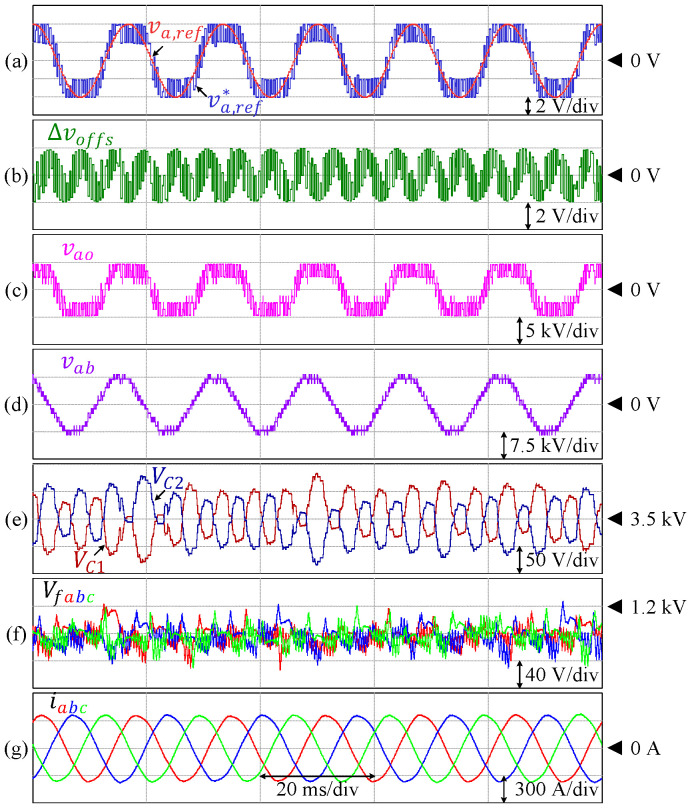
Performance of 9L-HCHB inverter at unity modulation index. (**a**) Reference voltages. (**b**) Offset voltage. (**c**) Pole voltage. (**d**) Line voltage. (**e**) Split DC-link capacitor voltages. (**f**) Flying capacitor voltages. (**g**) Output currents.

**Figure 9:**
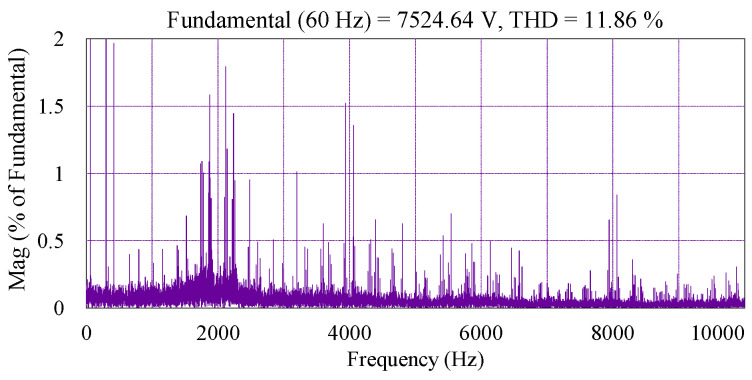
Harmonic spectrum of line voltage of 9L-HCHB inverter at unity modulation index.

The performance of a 9L-HCHB inverter during a steady state in the unity modulation index is shown in Figure 8, where both pole and line voltages exhibit the maximum number of levels. In order to maintain the capacitor voltages, the reference voltage at each phase leg ($v_{x,ref}$) is injected with the offset voltage (${∆v}_{offs}$), and thus results in the generation of a new reference voltage ($v_{x,ref}^{*}$). DC-link and flying capacitor voltages are regulated at 3500 V and 1166.67 V, respectively, where the maximum ripples are 2.37% and 5.07% of the references, respectively. The harmonic spectrum of line voltage is shown in Figure 9, where the THD is 11.86%.

**Figure 10:**
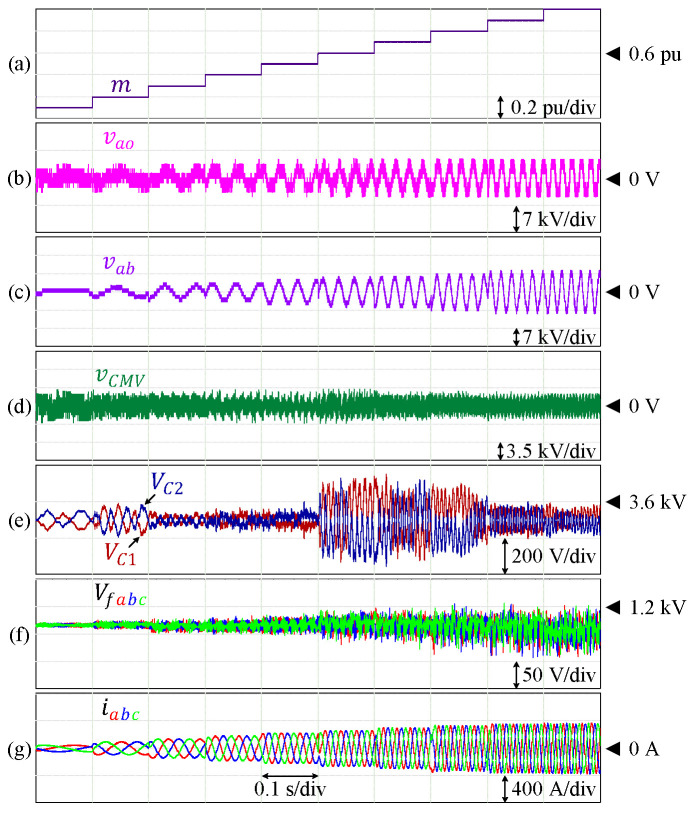
Performance of 9L-HCHB inverter at various modulation indices. (**a**) Modulation index. (**b**) Pole voltage. (**c**) Line voltage. (**d**) Common-mode voltage. (**e**) Split DC-link capacitor voltages. (**f**) Flying capacitor voltages. (**g**) Output currents.

As discussed earlier, the flying capacitor voltages are expected to drift at certain modulation indices under the conventional scheme. Figure 10 shows the performance of this 9L-HCHB inverter at various amplitude and frequency modulation indices ($m_{a}$ = $m_{f}$ = m) under the proposed balancing control. Each of the DC-link and flying capacitors is regulated at the corresponding reference, where the maximum voltage ripples are 7.21% and 5.06% of the references, respectively.

**Figure 11:**
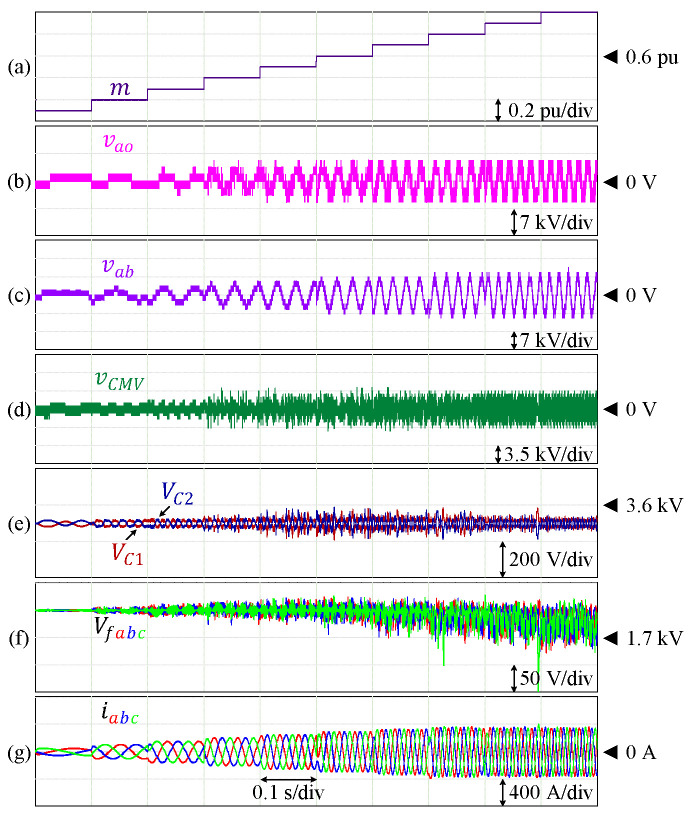
Performance of 7L-HCHB inverter at various modulation indices. (**a**) Modulation index. (**b**) Pole voltage. (**c**) Line voltage. (**d**) Common-mode voltage. (**e**) Split DC-link capacitor voltages. (**f**) Flying capacitor voltages. (**g**) Output currents.

**Figure 12:**
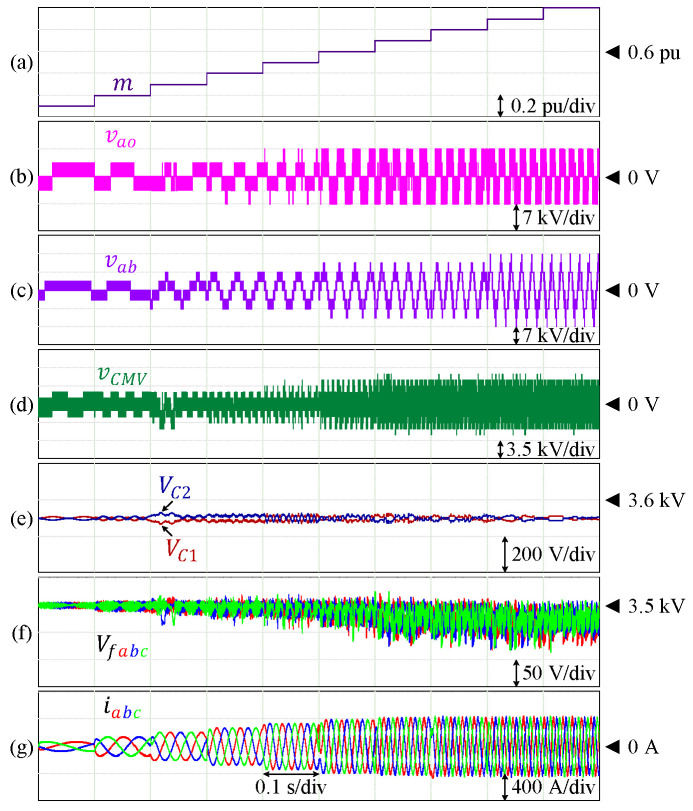
Performance of 5L-HCHB inverter at various modulation indices. (**a**) Modulation index. (**b**) Pole voltage. (**c**) Line voltage. (**d**) Common-mode voltage. (**e**) Split DC-link capacitor voltages. (**f**) Flying capacitor voltages. (**g**) Output currents.

The effectiveness of this control technique has also been verified for other HCHB inverters with a lower number of levels. Figure 11 shows the performance of a 7L-HCHB inverter under various modulation indices, where the voltages of the DC-link and flying capacitors are maintained at 3500 V and 1750 V, respectively. The maximum voltage ripples of the DC-link capacitors are 2.49% of the reference, whereas those of the flying capacitors are 8.62% of the reference, respectively. A similar simulation has also been conducted for a 5L-HCHB inverter as shown in Figure 12, where each capacitor voltage is maintained at 3500 V. The maximum voltage ripples at the DC-link and flying capacitors are 0.93% and 2.33% of the references, respectively.

The common-mode voltages ($v_{CMV}$) at HCHB inverters with a lower number of levels are higher due to the higher amplitude of pole voltages and voltage steps (dv/dt). The fluctuation of $v_{CMV}$ in 9L-HCHB, 7L-HCHB, and 5L-HCHB inverters are presented in Figure 10d–Figure 12d, where the amplitudes are 46.40%, 58.71%, and 83.29% of $V_{dc}$, respectively. Meanwhile, the root-mean-square (RMS) values are 16.74%, 20.25%, and 36.02% of $V_{dc}$, respectively.

**Figure 13:**
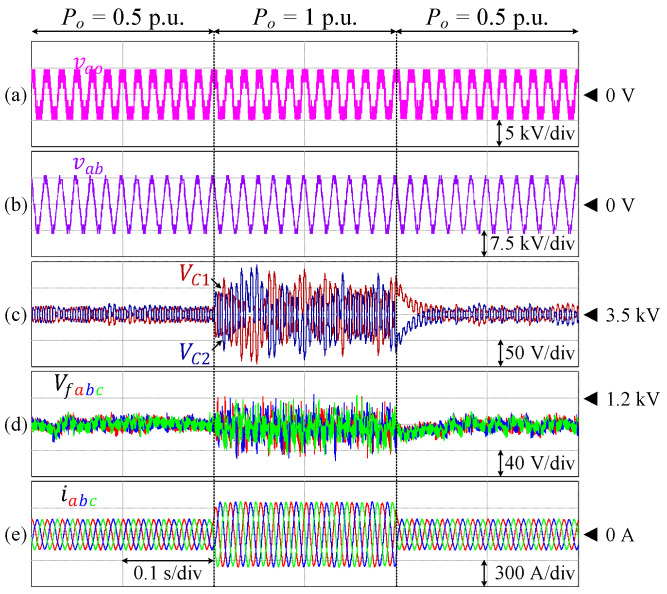
Performance of 9L-HCHB inverter under dynamic load changes. (**a**) Pole voltage. (**b**) Line voltage. (**c**) Split DC-link capacitor voltages. (**d**) Flying capacitor voltages. (**e**) Output currents.

The proposed control technique has also been verified for operation under dynamic load changes, as presented in Figure 13. In this case, the load of a 9L-HCHB inverter varies between 50% of the rated load ($P_{o}$ = 0.53 p.u. = 1.03 MW) and the rated load ($P_{o}$ = 1 p.u. = 1.94 MW). Each of the DC-link and flying capacitor voltages manage to be controlled with low fluctuation, where the maximum voltage ripples are 2.67% and 4.90% of the references, respectively.

**Figure 14:**
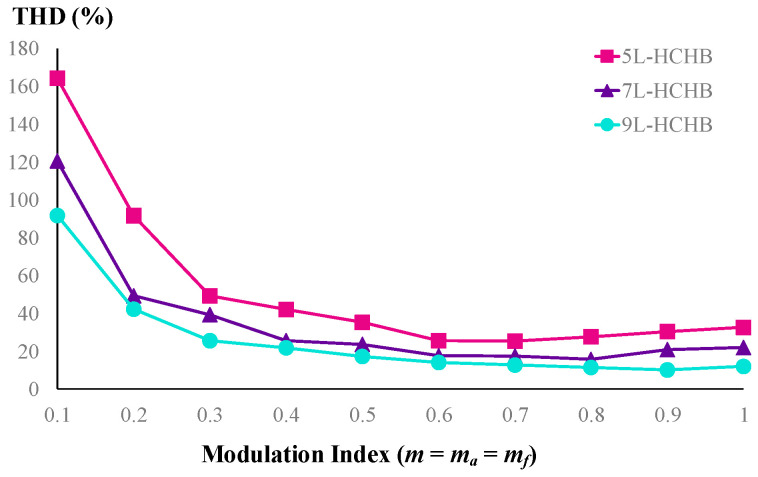
THD of line voltages at odd-level HCHB inverters.

A comparison of the THD values of line-to-line voltages at these odd-level HCHB inverters under various modulation indices is depicted in Figure 14. Since the inverter with a higher number of levels generates lower dv/dt and more sinusoidal voltage, the THD values are also lower at all modulation indices. For instance, the THD of five-level, seven-level, and nine-level HCHB inverters under unity modulation index are 32.51%, 21.89%, and 11.86%, respectively.

**Figure 15:**
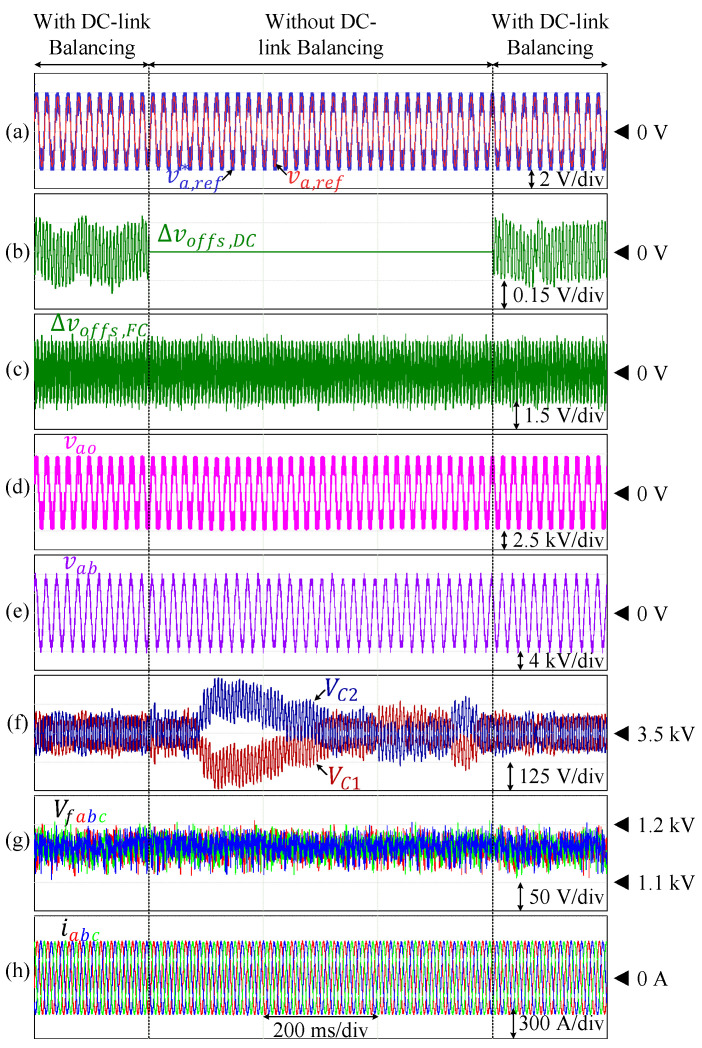
Performance of the inverter under the conventional scheme without offset injection for split DC-link voltage balancing and under the proposed scheme. (**a**) Reference voltages. (**b**) Injected offset for split DC-link voltage control. (**c**) Injected offset for FC voltage control. (**d**) Pole voltage. (**e**) Line-to-line voltage. (**f**) Split DC-link capacitor voltages. (**g**) Flying capacitor voltages. (**h**) Output currents.

**Figure 16:**
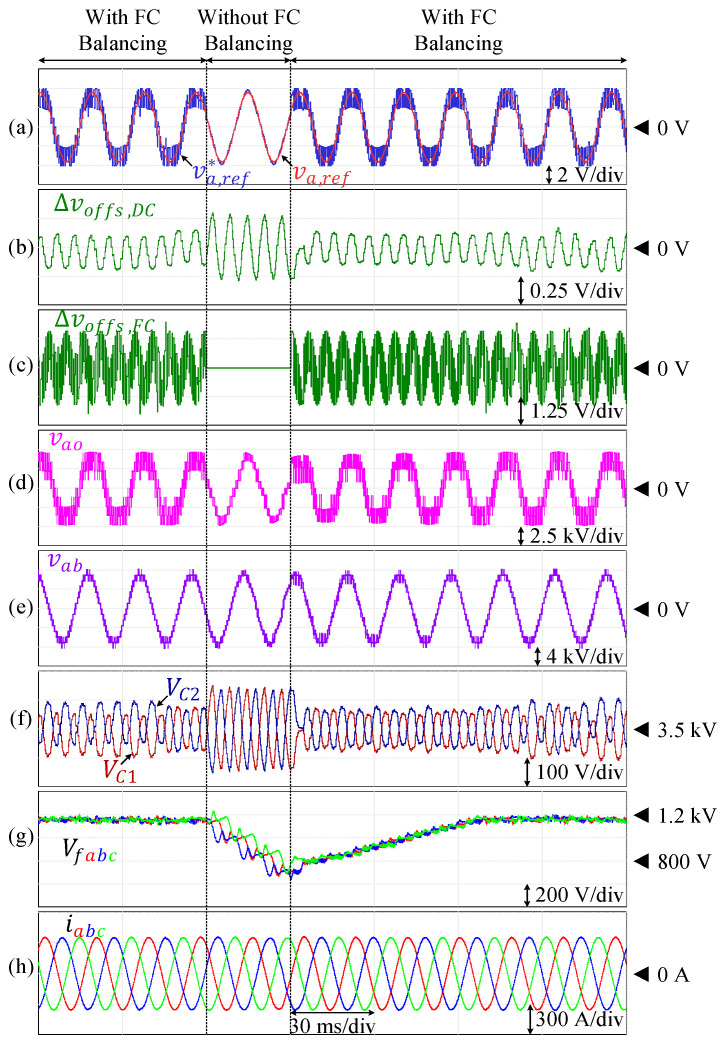
Performance of the inverter under the conventional scheme without offset injection for flying capacitor voltage balancing and under the proposed scheme. (**a**) Reference voltages. (**b**) Injected offset for split DC-link voltage control. (**c**) Injected offset for FC voltage control. (**d**) Pole voltage. (**e**) Line-to-line voltage. (**f**) Split DC-link capacitor voltages. (**g**) Flying capacitor voltages. (**h**) Output currents.

As discussed earlier, the split DC-link capacitor voltages are theoretically capable of self-balancing due to the symmetry in the polarity of phase current over one fundamental cycle. As a result, deactivating the corresponding voltage control (${∆v}_{offs,DC}=0$) does not significantly affect the voltage balance of $V_{C1}$ and $V_{C2}$, as demonstrated in Figure 15, where the DC-link capacitor voltages are naturally balanced. Nevertheless, the implementation of this closed-loop control is still applied to mitigate voltage drifts in the real environment with dynamic conditions. On the other hand, when the flying capacitor voltage balancing is deactivated (${∆v}_{offs,FC}=0$) during an operation in an unbalanced area, each of the flying capacitor voltages deviates from the reference value. This is demonstrated in Figure 16, where the inverter is operated at PF = 0.83 and $m_{a}=0.9$. Deactivation of the corresponding offset voltage leads to deviation of each flying capacitor voltage from the reference value. These capacitor voltages are restored back to the reference value as soon as the offset voltage is reintroduced to the modulation voltage reference.

To analyze the switching and conduction losses across the power switches at various modulation indices, a series of simulations have been conducted using thermal modules in PSIM. The DIM800XSM45-TS001 modules (4500 V/800 A) (Manufacturer: Dynex Semiconductor Ltd. City, Country: Doddington Road, Lincoln, Lincolnshire, LN6 3LF, United Kingdom.)have been employed to simulate devices within all cascaded submodules of each HCHB inverter and the H-bridge submodules of the 5L-HCHB inverter. Simultaneously, the switches in the H-bridge submodules of 9L-HCHB and 7L-HCHB inverters have been modeled using DIM800XSM33-F000 (3300 V/800 A) and DIM800FSM17-A000 (1700 V/800 A), respectively.

**Figure 17:**
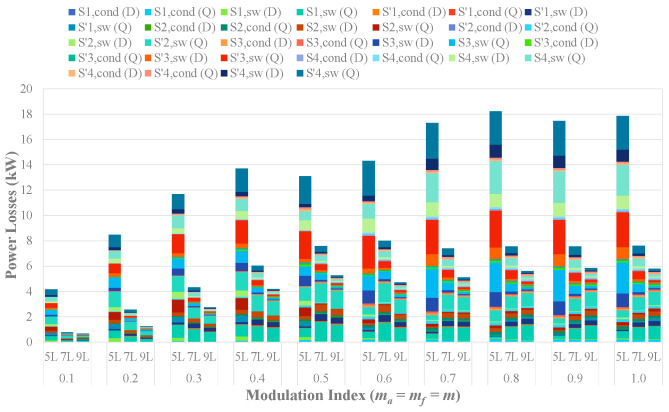
Switching and conduction losses across the power switches at various modulation indices.

Figure 17 illustrates the switching loss (“*sw*”) and conduction loss (“*cond*”) at the transistor (“*Q*”) and anti-parallel diode (“*D*”) of each power switch. The results reveal that lower switching and conduction losses are dissipated in inverters with a higher number of levels. At the unity modulation index, the power losses for the 5L-HCHB, 7L-HCHB, and 9L-HCHB inverters are 17.9 kW, 7.6 kW, and 5.8 kW, respectively.

**Figure 18:**
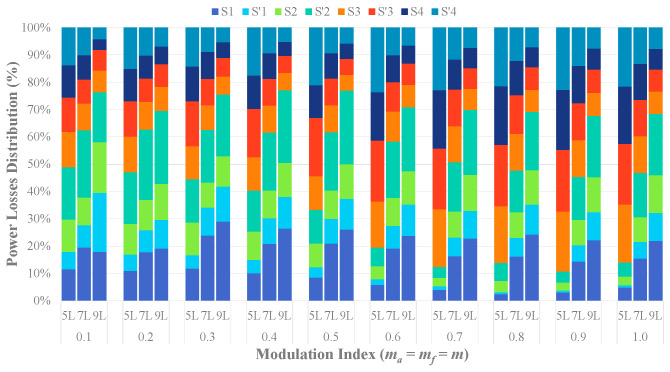
Power losses distribution across the switches.

Figure 18 illustrates the power loss distribution across the switches. In the 5L-HCHB inverter, the highest power dissipations are contributed by the H-bridge submodules, which contribute up to 89% of the total power losses. The opposite trend is exhibited in the 9L-HCHB inverter at all modulation indices, where the cascaded submodules contribute up to 77% of the total power losses. Meanwhile, the power losses in the 7L-HCHB inverters are more evenly distributed between both submodules at high modulation indices and uniformly distributed across the switches in the H-bridge submodules.

## 5. Experimental Verifications

**Figure 19:**
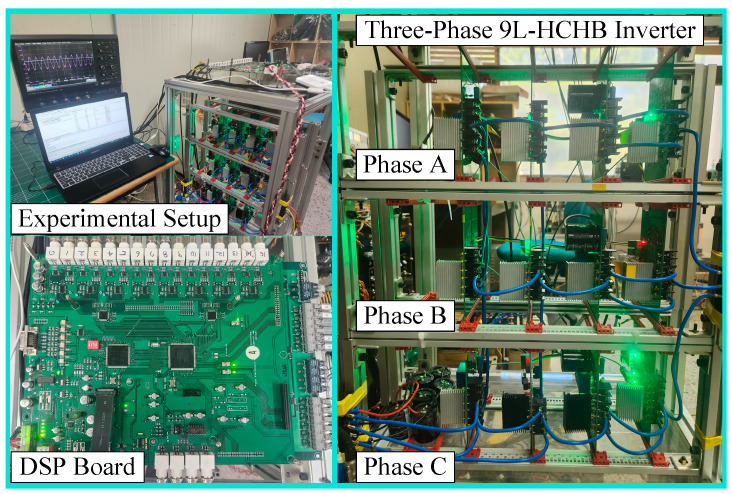
Experimental prototype of 9L-HCHB inverter.

In order to further validate the proposed operating scheme, a down-scaled three-phase 9L-HCHB inverter has been developed and operated with the parameters listed in Table 6. This prototype is controlled with a DSP chip (TMS320F28335)( Manufacturer: Texas Instruments Incorporated City, country: 12500 TI Blvd., Dallas, Texas 75243 USA) and a field-programmable gate array (Xilinx XC3S400) (Manufacturer: Xilinx, Inc., City, country: San Jose, CA, USA), as shown in Figure 19.

**Figure 20:**
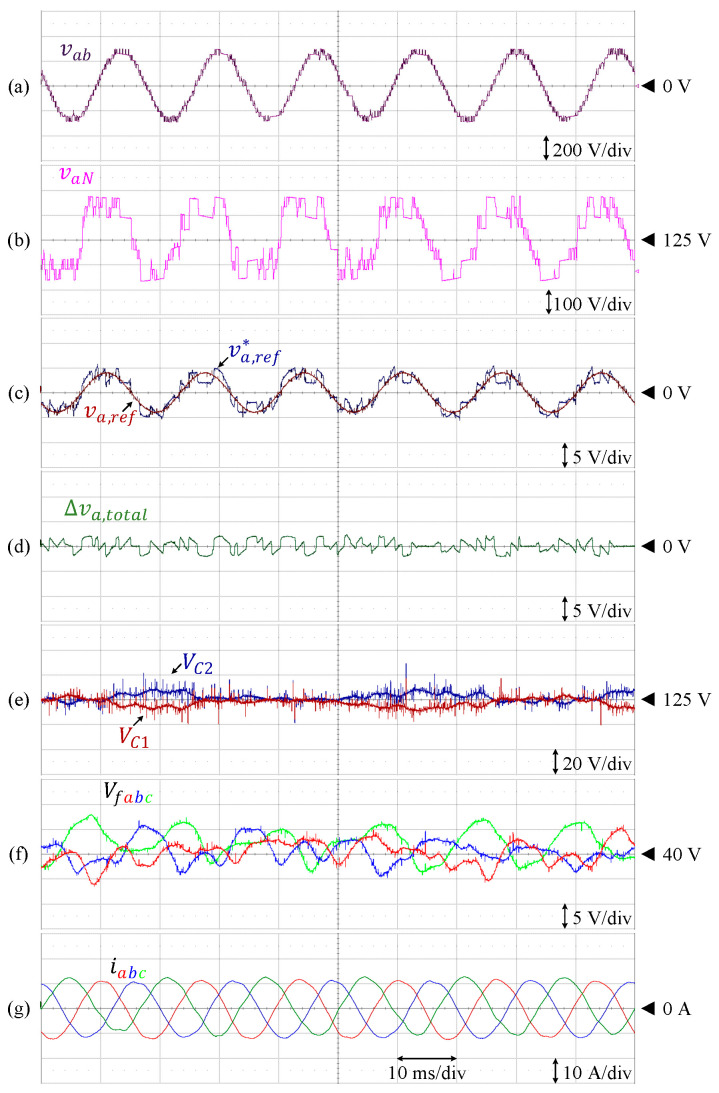
Performance of 9L-HCHB inverter prototype at unity modulation index. (**a**) Line voltage. (**b**) Pole voltage. (**c**) Reference voltages. (**d**) Injected offset voltage. (**e**) Split DC-link capacitor voltages. (**f**) Flying capacitor voltages. (**g**) Output currents.

**Figure 21:**
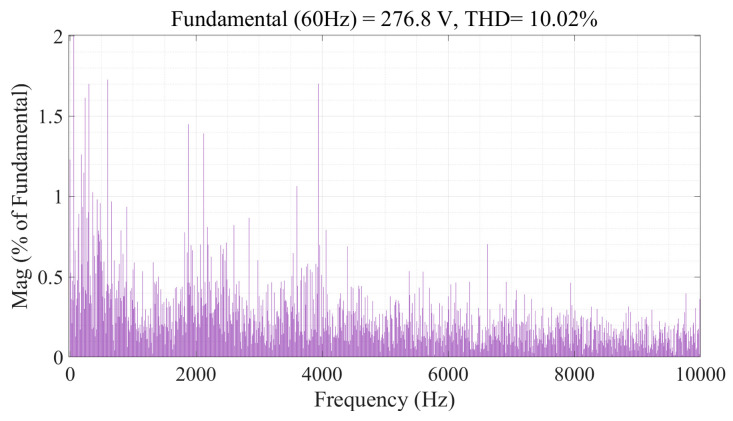
Harmonic spectrum of line voltage of 9L-HCHB inverter prototype at unity modulation index.

The steady-state performance of this inverter at unity modulation index is presented in Figure 20. The DC-link and flying capacitor voltages are controlled at 125 V and 41.67 V, respectively, where the peak-to-peak voltage ripples are kept within the allowable range, i.e., 20% of the corresponding references. The harmonic spectrum of the line-to-line voltage is depicted in Figure 21, where the THD is 10.02%. The harmonic components constituting this voltage also resemble that of the simulation.

**Figure 22:**
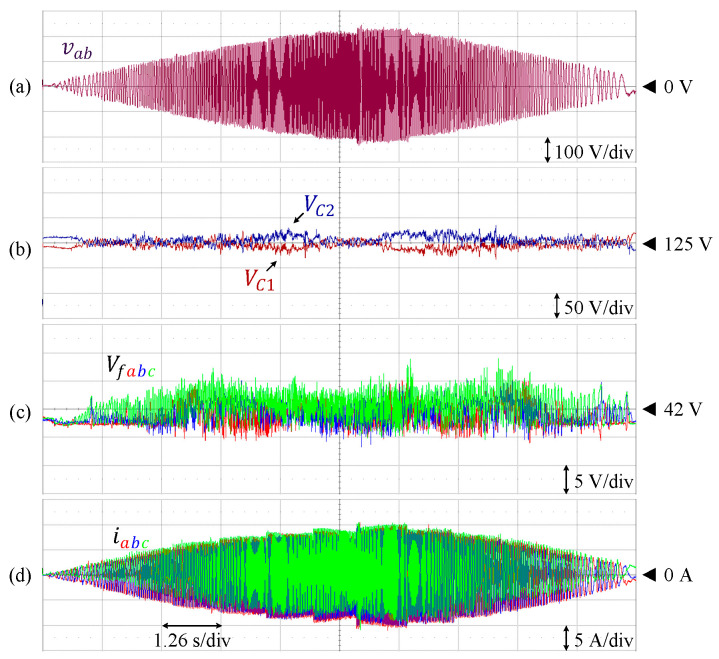
Performance of 9L-HCHB inverter prototype at various modulation indices. (**a**) Line voltage. (**b**) Split DC-link capacitor voltages. (**c**) Flying capacitor voltages. (**d**) Output currents.

The proposed control technique is also effective to maintain each of the DC-link and flying capacitor voltages at various modulation indices, as shown in Figure 22. Both the amplitude and modulation indices are gradually increased from m = 0 to m = 1 before both parameters are reduced back to m = 0. In this case, each of the DC-link and flying capacitor voltages are controlled at the references, and the fluctuations are kept within the allowable range.

**Figure 23:**
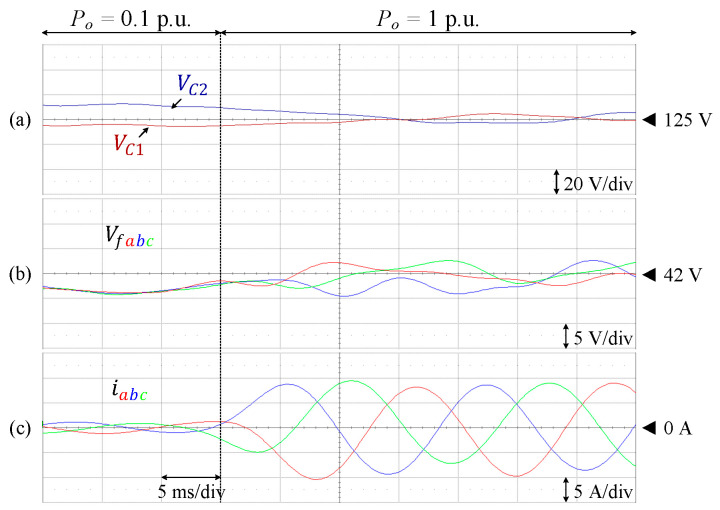
Performance of 9L-HCHB inverter prototype for step load changes. (**a**) Split DC-link capacitor voltages. (**b**) Flying capacitor voltages. (**c**) Output currents.

Figure 23 shows the transient state responses for load variation, where the load is increased from 12.7% ($P_{o}$ = 318 W) to 100% of the rated value ($P_{o}$ = 2.5 kW). Each of the DC-link and flying capacitor voltages is also maintained at the corresponding reference.

## 6. Conclusions

In this paper, a novel control scheme has been proposed to generalize the operating technique of hybrid cascaded H-bridge (HCHB) inverters and counter the capacitor voltage drifts at certain modulation indices. This is achieved by injecting two independent offset voltages to the modulation voltage references. The first offset voltage is obtained through a proportional control based on the deviation at the DC-link capacitor voltages. Meanwhile, the second offset voltage is generated by forcing the common reference to the nearest levels in the same cluster with opposing charging characteristics that compensate for the lack of switching state redundancy for balancing each flying capacitor voltage. The effectiveness of the proposed technique in simultaneously maintaining all capacitor voltages at the full range of operating conditions and within the allowable fluctuation range has been verified through simulation and experimental results.

## Figures and Tables

**Table 1:** Generalized switching states pattern for HCHB inverters.

$S_{x1}$	$S_{x2}$	$S_{x3}$	$S_{x4}$	$v_{xN}$	$i_{C1,x}$	$i_{C2,x}$	$i_{fx}$
1	1	0	1	$V_{C1}+V_{C2}+V_{fx}$	↑	↑	↑
1	1	1	1	$V_{C1}+V_{C2}$	↑	↑	−
1	1	0	0	$V_{C1}+V_{C2}$	↑	↑	−
1	1	1	0	$V_{C1}+V_{C2}-V_{fx}$	↑	↑	↓
0	1	0	1	$V_{C2}+V_{fx}$	−	↑	↑
0	1	1	1	$V_{C2}$	−	↑	−
0	1	0	0	$V_{C2}$	−	↑	−
0	1	1	0	$V_{C2}-V_{fx}$	−	↑	↓
0	0	0	1	$V_{fx}$	−	−	↑
0	0	1	1	0	−	−	−
0	0	0	0	0	−	−	−
0	0	1	0	$-V_{fx}$	−	−	↓

−: No current flows through the corresponding capacitor $\left(i_{x}>0\right)$. ↑: Current discharges the corresponding capacitor $\left(i_{x}>0\right)$. ↓: Current charges the corresponding capacitor $\left(i_{x}>0\right)$.

**Table 2:** Comparison of various HCHB inverters.

Number of Level	Base Voltage (E)	$V_{C1}$	$V_{C2}$	$V_{fx}$	TSV
5-level	$V_{dc}/2$	E	E	E	$12V_{dc}$
6-level *	$V_{dc}/3$	E	2E	E	$10V_{dc}$
		2E	E		
7-level	$V_{dc}/4$	2E	2E	E	$9V_{dc}$
8-level *	$V_{dc}/5$	2E	3E	E	$8.4V_{dc}$
		3E	2E		
9-level	$V_{dc}/6$	3E	3E	E	$12V_{dc}$

* Even level inverters possess two configuration options.

**Table 3:** Common capacitor voltage balancing methods for multilevel inverters.

Balancing Method	Advantages	Drawbacks
Phase-shifted carrier PWM [20,55]	Simplicity in implementation Direct control over the modulation index	Limited balancing capability under unbalanced loads Higher complexities at higher number of levels
Nearest level control [56]	Straightforward to implement	May not achieve perfect voltage balance
Zero-sequence voltage injection [57]	Effective for balancing capacitor voltage	Additional complexity in control implementation
Selective harmonic elimination [58]	Balancing through harmonic injection	Higher complexities at higher number of levels
Model predictive control [59]	Capable of handling constraints and providing optimal control	High computational demands, not real time friendly
Fuzzy logic control [60]	Adaptive control based on fuzzy rules	Tuning complexity May require extensive parametrization
Auxiliary balancing circuit [32]	Providing higher output voltage quality at the inverter due to the reduced burden of capacitor voltage balancing	Increased device count and the overall volume of the system

**Table 4:** Offset injection for regulating flying capacitor voltages in 9L-HCHB inverter.

$\mathrm{Cluster}\;\mathrm{of}\;v_{cm}$	Condition	${∆v}_{offs,FC}$	
		$i_{cm}\geq 0$	$i_{cm}<0$
All	$\left|{∆V}_{f,max}\right|\leq \varepsilon_{FC}E$	0	0
Cluster I ($2\leq v_{cm}\leq 4$)	${∆V}_{f,max}>\varepsilon_{FC}E$	$4-v_{cm}$	$2-v_{cm}$
	${∆V}_{f,max}<-\varepsilon_{FC}E$	$2-v_{cm}$	$4-v_{cm}$
Cluster II ($0\leq v_{cm}<2$)	${∆V}_{f,max}>\varepsilon_{FC}E$	$1-v_{cm}$	$2-v_{cm}$
	${∆V}_{f,max}<-\varepsilon_{FC}E$	$2-v_{cm}$	$1-v_{cm}$
Cluster III ($-2\leq v_{cm}<0$)	${∆V}_{f,max}>\varepsilon_{FC}E$	$-2-v_{cm}$	$-1-v_{cm}$
	${∆V}_{f,max}<-\varepsilon_{FC}E$	$-1-v_{cm}$	$-2-v_{cm}$
Cluster IV ($-4\leq v_{cm}\leq -2$)	${∆V}_{f,max}>\varepsilon_{FC}E$	$-2-v_{cm}$	$-4-v_{cm}$
	${∆V}_{f,max}<-\varepsilon_{FC}E$	$-4-v_{cm}$	$-2-v_{cm}$

**Table 5:** Parameters for simulation.

Parameters	Symbol	Value
DC-bus voltage	$V_{dc}$	7000 V
DC-link capacitors	$C_{1}$ $,\;C_{2}$	2.7 mF (5L)/1.35 mF (7L)/0.9 mF (9L)
Flying capacitors	$C_{fx}$	2.7 mF (all levels)
Fundamental frequency	$f_{0}$	60 Hz
Carrier frequency	$f_{sw}$	2000 Hz
*RL*-load	R , L	$10\;Ω,\;18\;\mathrm{mH}\;(P_{o}$ = 1 p.u. = 1.94 MW)

**Table 6:** Parameters for experiment.

Parameters	Symbol	Value
DC-bus voltage	$V_{dc}$	250 V
DC-link capacitors	$C_{1}$ $,\;C_{2}$	0.9 mF
Flying capacitors	$C_{fx}$	2.7 mF
Fundamental frequency	$f_{0}$	60 Hz
Carrier frequency	$f_{sw}$	2000 Hz
*RL*-load	R , L	$10\;Ω,\;18\;\mathrm{mH}\;(P_{o}$ = 2.5 kW)

## Data Availability

Data are contained within the article.
